# The Protective Effect of *Adenocaulon himalaicum* Edgew. and Its Bioactive Compound Neochlorogenic Acid against UVB-Induced Skin Damage in Human Dermal Fibroblasts and Epidermal Keratinocytes

**DOI:** 10.3390/plants10081669

**Published:** 2021-08-13

**Authors:** Hye Shin Ahn, Hyun Jae Kim, Changseon Na, Dae Sik Jang, Yu-Kyong Shin, Sun Hee Lee

**Affiliations:** 1 New Material Development Team, COSMAX BIO Ltd., 255 Pangyo-ro, Bungdang-gu, Seongnam 13486, Gyeonggi-do, Korea; hsahn@cosmax.com (H.S.A.); hyounjeakim@cosmax.com (H.J.K.); csna@cosmax.com (C.N.); 2 Department of Life and Nanopharmaceutical Sciences, Graduate School, Kyung Hee University, Seoul 02447, Dongdaemun-gu, Korea; dsjang@khu.ac.kr

**Keywords:** *Adenocaulon himalaicum* Edgew., neochlorogenic acid, UVB, MAPK, AP-1, filaggrin, photoaging

## Abstract

Skin aging induced by ultraviolet (UV) irradiation increases expression of matrix metalloproteinase-1 (MMP-1) and destroys collagen fibers, as a result accelerating wrinkle formation. Natural products have been received scientific attention as utilized agents against photoaging. The aim of this study was to investigate the protective effect of *Adenocaulon himalaicum* Edgew. extract (AHE) against ultraviolet B (UVB)-induced skin damage, and to explain the underlying mechanisms in human dermal fibroblasts and epidermal keratinocytes. AHE effectively protects skin photoaging by preventing collagen degradation through MMP-1 inhibition via the MAPK/AP-1 signaling pathway. AHE significantly increased the expression of skin hydration factors, such as filaggrin, involucrin, loricrin, and caspase-14. To find how AHE possesses a direct impact on cellular activities, we identified neochlorogenic acid as a bioactive component of AHE for the first time. Neochlorogenic acid showed the anti-photoaging effect through ameliorating UVB-induced collagen degradation, reinforcing the skin barrier. Like the AHE-regulating mechanism, neochlorogenic acid modulates the MAPK/AP-1 signaling pathway and skin hydration factors. Taken together, these results suggest that AHE and neochlorogenic acid are well-qualified candidate for enhancing the conditions of photoaged skin.

## 1. Introduction

Skin is the largest organ of human body which performs the essential barrier functions from water loss, chemical damages, infections, and ultraviolet radiation. Skin aging is the cumulative result of intrinsic and extrinsic aging. Intrinsic aging, often called chronological aging, is the inevitable physiological process that results from the age-dependent decline of cell function. On the other hand, extrinsic aging refers to a phenomenon in which progresses due to external factors such as ultraviolet exposure, environmental pollution, smoking, and drinking [1].

Particularly, skin aging induced by ultraviolet (UV) irradiation causes wrinkles, impaired pigmentation, shallowness, roughness, and diverse changes in fibroblasts and keratinocytes. UV radiation activates the mitogen-activated protein kinases (MAPKs) including extracellular signal-regulated kinase (ERK), p38 kinase, and c-Jun N-terminal kinase (JNK). MAPKs proteins lead to the upregulation of the AP-1 complex (c-Fos and c-Jun) transcription factors. Furthermore, increased AP-1 activity stimulates expression of matrix metalloproteinases (MMPs) that leads to collagen degradation, finally enhancing the skin photoaging [2].

Skin aging is also associated with the loss of a skin barrier function in epidermis. In the differentiation of keratinocytes, filaggrin, involucrin, and loricrin are expressed to aggregate with keratin filaments, which form a cornified cell envelope to protect the skin from environmental damages. The cornified cell envelope is responsible for maintaining skin hydration and water retention within the stratum corneum, the outermost layer of the epidermis [3,4,5]. The natural moisturizing factors (NMF) are formed from profilaggrin proteolysis to filaggrin, then finally from small hygroscopic molecules, including amino acids. These further degraded filaggrin monomers by caspase-14 are NMF, which are important in maintaining skin hydration and a low pH [6]. Notably, numerous studies have documented that ultraviolet (UV) light are the major causes for disruption of the epidermal barrier function, accompanied by a decrease in keratin, filaggrin, and involucrin levels. Therefore, the impaired skin barrier function promotes skin photoaging [7].

*Adenocaulon himalaicum* Edgew., also known as Asian trailplant, is a native plant to Ulleung-do, Korea. According to “*Traditional Knowledge and Use of Folk Plants in Korea*” published by the National Arboretum of the Korea Forest Service, *A. himalaicum* is traditionally known to be eaten as a vegetable by boiling the leaves or making a soup. In addition, various parts of *A. himalaicum*, including the leaves, seeds, roots, and stems, have been used as ingredients in traditional medicine for treating abscesses, hemorrhage, and inflammation [8]. Previous studies with *A. himalaicum* have not proven its biological effects except antioxidant [9] and chemopreventive effect [10].

The anti-photoaging activities of *A. himalaicum* have not been reported yet. In this study, we demonstrated the protective effect of *A. himalaicum* extract in UVB-induced human dermal fibroblasts (HS68) and human epidermal keratinocytes (HaCaT) cells through analysis of MMP-1 production, protein kinase (ERK, JNK and p38), and downstream molecules. In addition, we isolated the component from *A. himalaicum* and determined its anti-photoaging properties in UVB-irritated skin cells.

## 2. Results

### 2.1. The Effects of AHE on Cell Proliferation, MMP-1 and Procollagen Type I Production in UVB-Irradiated Hs68 Fibroblasts

**Figure 1:**
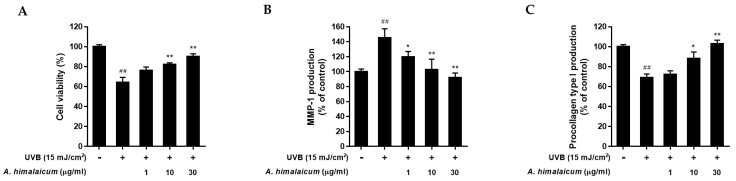
Effect of *Adenocaulon himalaicum* Edgew. (AHE) on cell proliferation, MMP-1 production and procollagen type I production in UVB-irradiated Hs68 fibroblasts. Cells were exposed to UVB at 15 mJ/cm^2^ and then treated with AHE (1, 10, 30 μg/mL) for 24 h. (**A**) Cell viability was measured by an MTT assay. (**B**) The cell culture media were collected to examine the levels of MMP-1 using an ELISA KIT. (**C**) The cell culture media were collected to examine the levels of procollagen type I using an ELISA KIT. Results are expressed as mean ± S.D. of three independent experiments; ^##^ *p* < 0.01 compared with the non-UVB-irradiated control; * *p* < 0.05, ** *p* < 0.01 compared with the UVB-irradiated control.

First, we evaluated the cytoprotective effect of AHE in UVB-irradiated Hs68 fibroblasts using an MTT assay. As shown in Figure 1A, UVB exposure reduced cell viability by 64.25% ± 2.13% compared to the control cells. However, AHE diminished the cytotoxicity induced by UVB (76.12% ± 3.6 7% at 1 μg/mL, 82.59% ± 1.75% at 10 μg/mL and 90.29% ± 2.85% at 30 μg/mL, respectively). Then, we investigated the effect of AHE treatment on MMP-1 and procollagen type I protein expression. AHE significantly reduced the UVB-induced MMP-1 production (Figure 1B), suggesting that the AHE could inhibit the collagen degradation. As a result of the increased MMP-1 expression, UVB radiation-exposed cells showed remarkably reduced procollagen type I secretion by 68.86% ± 3.78%. However, AHE treatment significantly improved the production of procollagen type I up to 102.60% ± 3.93% at 30 μg/mL (Figure 1C).

### 2.2. The Effects of AHE on MAPK and AP-1 Complex Signaling Pathways in UVB-Irradiated Hs68 Fibroblasts

**Figure 2:**
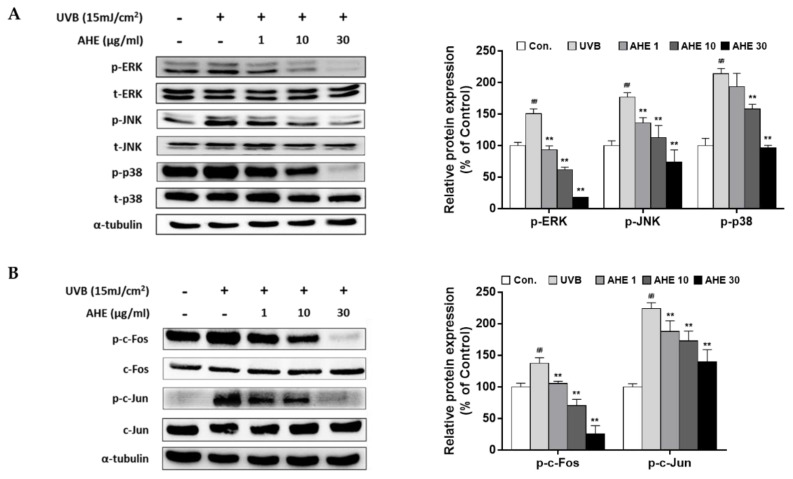
Effects of AHE on the mitogen-activated protein kinase (MAPK) and the activator protein 1 (AP-1) signaling pathway in UVB-irradiated Hs68 fibroblasts. Cells were exposed to UVB at 15 mJ/cm^2^ and then treated with AHE (1, 10, 30 μg/mL) for 1 h (MAPK) or 4 h (AP-1). Total protein was prepared and detected with specific (**A**) p-ERK, p-JNK, p-p38 antibodies or (**B**) p-c-Fos and p-c-Jun antibodies. Presented data are the representative blots of three independent experiments. The relative protein levels are expressed as mean ± S.D. (% of control) of three independent experiments; ^##^ *p* < 0.01 compared with the non-UVB-irradiated control; ** *p* < 0.01 compared with the UVB-irradiated control.

UVB irradiation has been reported to activate MAPKs and its downstream regulator, AP-1, thus accelerating MMP transcription [11]. Our results showed that UVB stimulated overall MAPK signaling molecules, but AHE suppressed the phosphorylation of extracellular signal-regulated kinase (ERK), c-Jun N-terminal kinases (JNK), and p38 (Figure 2A). Followed by MAPK signaling, UVB induced the phosphorylation of the AP-1 subunits (c-Fos and c-Jun), but AHE effectively inhibited theses effects (Figure 2B). Finally, the mRNA levels of MMP-1 were down-regulated by AHE treatment (Figure S3).

### 2.3. The Effect of AHE on mRNA Expression of Filaggrin, Involucrin, Loricrin, and Caspase-14 in UVB-Irradiated HaCaT Keratinocytes

**Figure 3:**
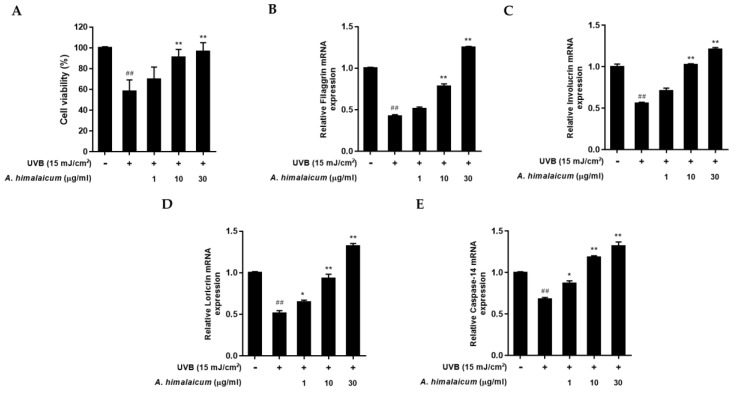
Effect of AHE on filaggrin, involucrin, loricrin, and caspase-14 mRNA expression levels in UVB-irradiated HaCaT keratinocytes. Cells were exposed to UVB at 15 mJ/cm^2^ and then treated with AHE (1, 10, 30 μg/mL) for 24 h. (**A**) Cell viability was evaluated with a MTT assay. (**B**–**E**) Total cellular RNA was extracted from AHE-treated cells. mRNA levels of filaggrin, involucrin, loricrin, and caspase-14 were quantified by qRT-PCR and adjusted to GAPDH. Results are expressed as mean ± S.D. of three independent experiments; ^##^ *p* < 0.01 compared with the non-UVB-irradiated control; * *p* < 0.05; ** *p* < 0.01 compared with the UVB-irradiated control.

To examine the effects of AHE on the skin barrier function in UVB-irradiated HaCaT keratinocytes, the mRNA level of filaggrin, involucrin, loricrin, and caspase-14 was measured by qRT-PCR analysis. We performed a MTT assay to find the cytoprotective effect of AHE in UVB-irradiated HaCaT keratinocytes. As shown in Figure 3A, UVB exposure reduced cell viability by 57.94% ± 11.18% compared to control cells; however, AHE lessened the cytotoxicity up to 96.59% ± 11.18% at 30 μg/mL treatment. Then, the regulations of filaggrin, involucrin, loricrin, and caspase-14 expression were evaluated. UVB irradiation reduced overall skin barrier proteins and caspase-14. However, AHE treatment up-regulated the expression of filaggrin, involucrin, and loricrin mRNA in a dose-dependent manner compared with UVB-irradiated control (Figure 3B–D). In addition, caspase-14 mRNA expression was also markedly increased by AHE treatment (Figure 3E), which indicated that AHE could contribute to recover the natural moisturizing factors.

### 2.4. Determination of Neochlorogenic Acid as a Constituent of AHE

**Figure 4:**
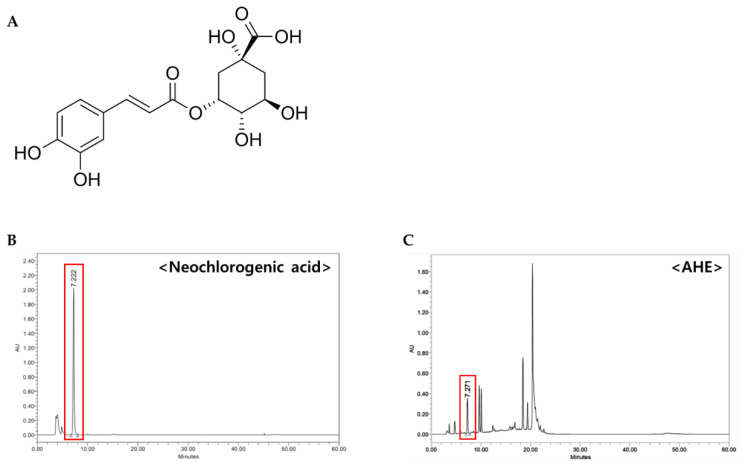
HPLC chromatograms of neochlorogenic acid and AHE detected by UV 325 nm. (**A**) Chemical structure of neochlorogenic acid isolated from AHE. (**B**) HPLC chromatograms of neochlorogenic acid and (**C**) AHE.

To search for bioactive compounds of AHE with anti-photoaging activity, we frac-tionated and isolated components from AHE. First, the dried leaves of *A. himalaicum* (1.1 kg) were extracted with 30% ethanol for 5 h and obtained AHE (240 g). Repeated column chromatography with AHE and its subfraction (2.82 g) using RP-MPLC were performed to yield neochlorogenic acid (Figure 4A). The structure of the neochlorogenic acid was identified by analysis of ^1^H-NMR and ^13^C-NMR spectra, and a comparison with previous study [12] was carried out, as follows (Figures S1 and S2).

^1^H-NMR (600 MHz, methanol-d4) δ: 7.59 (d, 1H, J = 15.8Hz, H-3), 7.05 (d, 1H, J = 2.0 Hz, H-2′), 6.94 (dd, 1H, J = 8.2, 2.0, Hz, H-6′), 6.77 (d, 1H, J = 8.2 Hz, H-5′), 6.31 (d, 1H, J = 15.8 Hz, H-2), 5.35 (m, 1H, Q-3), 4.15 (m,1H, Q-5), 3.64 (m, 1H, Q-4),2.21 (m, 1H, Q-2a), 2.14 (m, 2H, Q-6), 1.96 (m,1H, Q-2b).

^13^C-NMR (150 MHz, methanol-d4) δ: 178.47 (Q-7), 169.19 (C-1), 149.59 (C-4′), 146.96 (C-3′), 146.94 (C-3), 128.11 (C-1′), 123.03 (C-6′), 116.60 (C-5′), 115.98 (C-2), 115.23 (C-2′), 75.52 (Q-1), 74.94 (Q-5), 73.16 (Q-3), 68.42 (Q-4), 41.68 (Q-2), δ 36.85 (Q-6).

HPLC analysis was performed to determine the contents of neochlorogenic acid in AHE. As shown in the chromatogram, neochlorogenic acid was confirmed at 7.22 min (Figure 4B,C).

### 2.5. The Effects of Neochlorogenic Acid on Cell Proliferation, MMP-1 and Procollagen Type I Production in UVB-Irradiated Hs68 Fibroblasts

**Figure 5:**
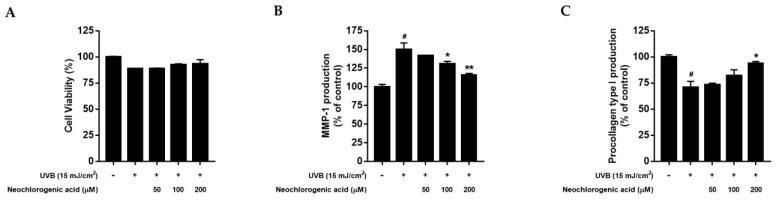
Effect of neochlorogenic acid on cell proliferation, MMP-1 production and procollagen type I production in UVB-irradiated Hs68 fibroblasts. Cells were exposed to UVB at 15 mJ/cm^2^ and then treated with neochlorogenic acid (50, 100, 200 μM) for 24 h. (**A**) Cell viability was measured by an MTT assay. (**B**) The cell culture media were collected to examine the levels of MMP-1 using an ELISA KIT. (**C**) The cell culture media were collected to examine the levels of procollagen type I using an ELISA KIT. Results are expressed as mean ± S.D. of three independent experiments; ^#^ *p* < 0.05 compared with the non-UVB irradiated control; * *p* < 0.05, ** *p* < 0.01 compared with the UVB-irradiated control.

To measure the cytotoxicity of neochlorogenic acid, an MTT assay was conducted in UVB-irradiated Hs68 fibroblasts. As shown in Figure 5A, UVB exposure reduced cell viability compared to control cells; however, neochlorogenic acid tended to diminish the decrease in cell proliferation and did not show the cytotoxicity up to 200 μM treatment. Then, we evaluated the effect of neochlorogenic acid treatment on MMP-1 and procollagen type I protein expression. As shown in Figure 5B, neochlorogenic acid treatment decreased the UVB-induced MMP-1 production. In addition, UVB irradiation markedly decreased the procollagen type I secretion, but neochlorogenic acid treatment significantly improved the production of procollagen type I up to 92.61% ± 1.93% at 200 μM (Figure 5C).

### 2.6. The Effects of Neochlorogenic Acid on MAPK and AP-1 Complex Signaling Pathways in UVB-Irradiated Hs68 Fibroblasts

**Figure 6:**
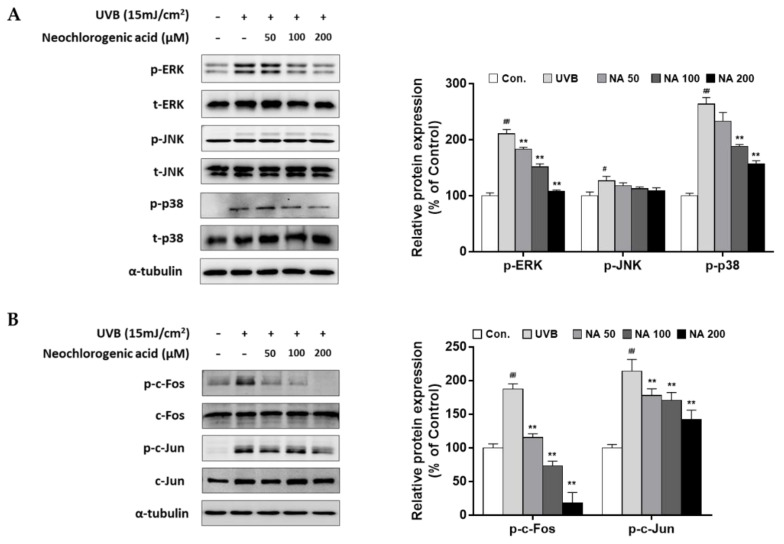
Effects of neochlorogenic acid on the mitogen-activated protein kinase (MAPK) and the activator protein 1 (AP-1) signaling pathway in UVB-irradiated Hs68 fibroblasts. Cells were exposed to UVB at 15 mJ/cm^2^ and then treated with neochlorogenic acid (50, 100, 200 μM) for 1 h (MAPK) or 4 h (AP-1). Total protein was prepared and detected with specific (**A**) p-ERK, p-JNK, p-p38 antibodies, or (**B**) p-c-Fos and p-c-Jun antibodies. Presented data are the representative blots of three independent experiments. The relative protein levels are expressed as mean ± S.D. (% of control) of three independent experiments; ^#^ *p* < 0.05; ^##^ *p* < 0.01 compared with the non-UVB irradiated control; ** *p* < 0.01 compared with the UVB-irradiated control.

To confirm the bioactivity of neochlorogenic acid as an active compound of AHE, upstream regulating molecules of MMP-1 expression were examined. As shown in Figure 6, our results showed that UVB stimulated overall MAPKs signaling molecules, but neochlorogenic effectively suppressed the phosphorylation of ERK and p38, but not JNK (Figure 6A). Followed by MAPK signaling, UVB induced the phosphorylation of the AP-1 subunits (c-Fos and c-Jun), and neochlorogenic acid remarkably inhibited theses effects (Figure 6B). Additionally, the mRNA levels of MMP-1 were down-regulated by neochlorogenic acid treatment (Figure S4).

### 2.7. The Effect of Neochlorogenic Acid on mRNA Expression of Filaggrin, Involucrin, Loricrin, and Caspase-14 in UVB-Irradiated HaCaT Keratinocytes

**Figure 7:**
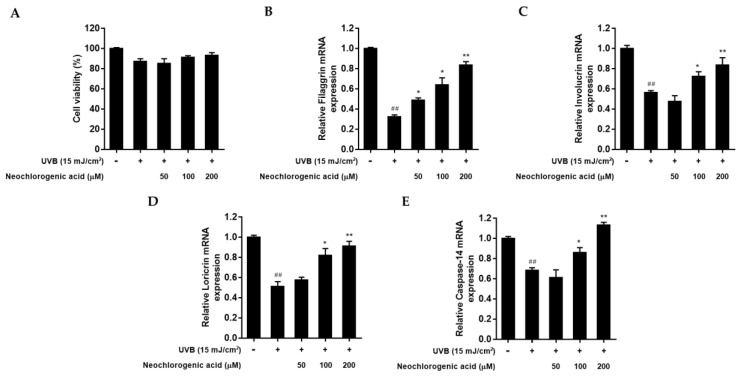
Effect of neochlorogenic acid on filaggrin, involucrin, loricrin, and caspase-14 mRNA expression levels in UVB-irradiated HaCaT keratinocytes. Cells were exposed to UVB at 15 mJ/cm^2^ and then treated with neochlorogenic acid (50, 100, 200 μM) for 24 h. (**A**) Cell viability was evaluated with an MTT assay. (**B**–**E**) Total cellular RNA was extracted from neochlorogenic acid-treated cells. mRNA levels of filaggrin, involucrin, loricrin, and caspase-14 were quantified by qRT-PCR and adjusted to GAPDH. Results are expressed as mean ± S.D. of three independent experiments; ^##^ *p* < 0.01 compared with the non-UVB-irradiated control; * *p* < 0.05, ** *p* < 0.01 compared with the UVB-irradiated control.

To examine whether neochlorogenic acid protects the skin barrier function from UVB radiation in HaCaT keratinocytes, the mRNA level of filaggrin, involucrin, loricrin, and caspase-14 was measured by qRT-PCR analysis. An MTT assay was conducted to investigate the cytotoxicity of neochlorogenic acid in UVB-irradiated HaCaT keratinocytes. As shown in Figure 7A, neochlorogenic acid did not affect cell viability up to 200 μM treatment. After UVB irradiation, the regulations of filaggrin, involucrin, loricrin, and caspase-14 expression were evaluated. UVB exposure reduced overall mRNA expressions. However, neochlorogenic acid treatment increased the expression of filaggrin, involucrin, loricrin, and caspase-14 mRNA in a dose-dependent manner compared with UVB-irradiated control (Figure 7B–E).

## 3. Discussion

As the largest organ of the body, the skin shows visible signs of aging when we are getting old. With increase in the average human lifespan, the dermatological needs of the aging skin become more important. Plant extracts have been known to antagonize UV-induced skin aging by the free-radical scavenging activity. Accordingly, the nutraceutical and cosmetic industries attempting to prevent or reverse skin aging have focused on numerous herbal ingredients. Therefore, in this report, we aimed to determine the protective activity of *Adenocaulon himalaicum* Edgew. (AHE) extract on skin photoaging and elucidate the associated molecular mechanisms.

UVB irradiation induces cytotoxicity by increasing reactive oxygen species (ROS) production, which stimulates the skin cell apoptosis [13]. Additionally, UVB-induced ROS subsequently activate complex signaling pathways, including MAPKs, AP-1, and MMPs, which finally degrade collagen fibers. MMP-1 is a major extracellular matrix component which cleaves collagen type I in the dermis [14]. Collagen type I is a major structural protein in the extracellular matrix of the dermis and is related to skin elasticity and moisture [15]. Therefore, decreased collagen deposition by UVB-induced MMP-1 causes skin photoaging. In our study, we confirmed that UVB irradiation induced the increase in MMP-1 expression and the decrease in procollagen type I. When AHE treated in UVB irradiated the fibroblast, it not only rescued the UVB-induced cytotoxicity, but also prevented against photoaging through the regulation of MMP-1 and procollagen I type expression.

The underlying mechanisms of collagen degradation in skin photoaging result from activating various transcriptional factors, including AP-1 and NF-κB [16]. The subunits of AP-1, c-Fos, and c-Jun are activated by a variety of stimuli, including UV irradiation. Many studies have demonstrated that the transcription and activation of c-Fos and c-Jun largely depends on the activation of the MAPK signaling molecules, which finally regulate the transcription of the collagen degradation enzymes, MMPs [17,18]. According to our results, AHE inhibited phosphorylation of ERK, JNK, and p-38, as well as phosphorylation of c-Fos and c-Jun AP-1 subunits. Therefore, AHE inhibited UVB-induced MMP-1 expression by the downregulation of MAPKs and the AP-1 signaling pathway.

Stratum corneum (SC) cells, the outermost layer of the epidermis, protect against skin dehydration and environmental hazards. Maintenance of an optimal level of hydration in the SC is largely dependent on the components of cornified cell envelope and natural moisturizing factors. Therefore, filaggrin, involucrin, loricrin, and caspase-14 are important sources for maintaining skin moisture [19,20]. In a previous study, downregulation of filaggrin and loricrin have been involved in skin reconstruction in vitro after UVB exposure [21]. In our study, mRNA expression of filaggrin, involucrin, loricrin, and caspase-14 was increased after AHE treatment, as compared to only UVB-exposed cells. These results showed that AHE could have positive effects on skin hydration and moisture retention.

Natural product extracts are a rich source of bioactive compounds, which exhibit the direct impact on physiological or cellular activities in the humans or animals that consume such compounds [22]. In previous studies, several constituents of AHE were identified and proved its anti-photoaging effects [23]. For example, β-sitosterol enhanced the expression of skin barrier functional proteins, including loricrin, filaggrin, and involucrin in keratinocytes [24]. Lupeol showed the inhibitory effect on MMPs expression through the down-regulation of the p-ERK pathway [25]. Oleanolic acid, also found in AHE, inhibited transient receptor potential vanilloid 1 channel activity [26].

In present study, we newly reported neochlorogenic acid as a component of AHE, which has never been identified, and studied its function on skin photoaging. Neochlorogenic acid is a natural polyphenolic compound found in dried fruits and other various sources of plants [27]. Neochlorogenic acid have functions on scavenging free radicals, antioxidant, anti-inflammatory, and antitumor activities [27,28]. It has already been established that a positive correlation between the antioxidant capacities prevents UV-induced skin damage [29], and that neochlorogenic acid is expected as a bioactive compound with anti-photoaging activity. When neochlorogenic acid was treated in a UVB-irradiated fibroblast, it regulated the MMP-1 and procollagen I type production through MAPKs and AP-1 signaling pathways. In addition, mRNA expression of filaggrin, involucrin, loricrin, and caspase-14 was increased by neochlorogenic acid treatment in UVB-irradiated keratinocytes. These results indicated that newly identified neochlorogenic acid is an active component of AHE with anti-photoaging effects in both the fibroblast and keratinocytes.

It is the first report to show the reliable potential of AHE to attenuate UVB-induced skin wrinkle formation and dehydration. In addition, for the first time, we discovered that neochlorogenic acid is the anti-photoaging agent and an active component that exists in AHE. To secure solid evidence of AHE and neochlorogenic acid for the remedy of skin aging, the UVB-induced animal model study and clinical trial for subjects with wrinkled and dried skin needs to be conducted. Those further studies would support that AHE should be recommended in prevention and treatment of skin anti-aging.

## 4. Materials and Methods

### 4.1. Preparation of A. himalaicum Extract

Leaves of *A. himalaicum* were collected from Ulleung-do, Korea. A specimen voucher was deposited in COSMAX BIO (COSMAX BIO Ltd., Seongnam, Korea). The dried leaves (1.1 kg) were extracted with 30% ethanol in a shaking water bath at 60 °C for 5 h. Then, the extract was filtered and concentrated using a vacuum rotary evaporator (Heidolph Instruments, schwabach, German) to obtain the *A. himalaicum* extract (AHE) (240 g) with a yield of 22.3% ± 0.5%.

### 4.2. Isolation and Characterization of Neochlorogenic Acid

Neochlorogenic acid was isolated from *A. himalaicum* extract (AHE). Briefly, 200 g of AHE was subjected to gel filtration using DIAION HP-20 resin. The developing solvent was [acetone: distilled water = 0:10 ~ 10:0] solvent fractionation using a mixed solution and divided into 12 small fractions (Fraction 1-1~12). Subfraction 4 was purified using RP-MPLC [acetonitrile: distilled water = 5:95 ~ 100:0] to obtain a pure single compound. The chemical structure was elucidated by instrumental analyses. The isolated compound was carried out by comparison of spectroscopical data. 1D-NMR experiments were conducted using ^1^H-(600 MHz) and ^13^C-(150 MHz) nuclear magnetic resonance (NMR) and recorded with AvanceIII-600 (Bruker, German). ^1^H-NMR spectra showed the existence of a caffeoyl moiety and three aromatic protons [δH 7.05 (d, 1H, J = 2.0 Hz, H-2′), 6.94 (dd, 1H, J = 2.0, 8.2 Hz, H-6′), 6.77 (d, 1H, J = 8.2 Hz, H-5′)] of the ABX spin system. Trans doublets [δH 7.59 (d, 1H, J = 15.8 Hz, H-3), 6.31 (d,1H, J = 15.8 Hz, H-2)] also showed the existence of a quinic acid moiety [δH 5.35 (m, 1H, Q-3), 4.15 (m, 1H, Q-5), 3.64 (m, 1H, Q-4), 2.21 (m, 1H, Q-2a), 2.14 (m, 2H, Q-6), 1.96 (m, 1H, Q-2b)]. ^13^C-NMR spectra showed the existence of sixteen carbon atoms consisting of two methylene, eight methine, and six quaternary carbon atoms, including two carbonyl groups [ δ 178.47 and δ 169.19]. The molecular weight was measured using a liquid chromatography mass spectrometer, and as a result, it was found to be C_16_H_18_O_9_ with 354.3 g/mol. As a result of structural identification of the compound, it was confirmed as neochlorogenic acid.

### 4.3. High-Performance Liquid Chromatography Analysis

AHE and neochlorogenic acid were analyzed by high-performance liquid chromatography (HPLC) and ultraviolet absorbance detector (UV/Vis detector). A HPLC instrument, a Waters e2695 Series system, and a Waters 24489 UV/Vis detector (Worcester, MA, USA), X select C18 (5 μm, 250 × 4.6 mm, waters, Milford, MA, USA) column were used. All HPLC-grade solvents purchased from Baker (Phillipsburg, NJ, USA) were used. For analysis, the temperature of the column was set to 35 °C, the injection volume was set to 20 μL, and the measurement wavelength was set to 325 nm. Acetonitrile (ACN), tertiary distilled water (DW), and formic acid were used as the mobile phase, and 0.1% formic acid ACN:0.1% formic acid DW (1:9–10:0, at a rate of 0.7 mL/min, *v/v*) in the mixed solution was analyzed for 60 min. For the analysis sample, 1 g of AHE was precisely weighed; 100 mL of 30% methanol was added, dissolved in an ultrasonic shaker for 30 min, and allowed to cool at room temperature; and the supernatant was filtered through a 0.22 μm membrane filter, and used. Neochlorogenic acid 1 mg was precisely weighed; 2 mL of 50% methanol was added, dissolved in an ultrasonic shaker for 30 min, and allowed to cool at room temperature; and methanol was added, and filtered through a 0.22 μm membrane filter. For each analysis, the chromatogram was extracted at 325 nm, and AHE and neochlorogenic acid peak were compared and analyzed.

### 4.4. Cell Culture

Human fibroblasts (Hs68) and human epidermal keratinocytes (HaCaT) were purchased from the American Type Culture Collection (ATCC, Manassas, VA, USA) and cultured in Dulbecco’s modified eagle’s medium (DMEM, Hyclone, Logan, UT, USA), supplemented with 10% fetal bovine serum (FBS), streptomycin, and penicillin in an atmosphere of 5% CO_2_ at 37 °C. Hs68 or HaCaT cells were plated in culture plate for 24 h. After incubation, the cells were washed once with Dulbecco’s phosphate-buffered saline (DPBS, Welgene, Gyeongsangbuk-do, Korea) and then exposed to UVB radiation using UVP crosslinker (Analytik Jena, Jena, Germany). To calculate the optimal UVB irradiation dose, Hs68 or HaCaT cell viability was determined using a 3-[4,5-dimethylthiazol-2-yl]-2, 5-diphenyltetrazolium bromide (MTT) assay after treatment of various doses of UVB radiation. Next, 15 mJ/cm^2^ of UVB radiation was chosen as the stimulation dose for further experiments. After UVB irradiation, cells were incubated in fresh culture media in the presence of AHE (1, 10, or 30 μg/mL) or neochlorogenic acid (50, 100 or 200 μM). 

### 4.5. Determination of Cell Viability

After UVB irradiation, Hs68 or HaCaT cells were treated with AHE or neochlorogenic acid for 24 h. Cell viability was measured using an MTT colorimetric assay (Sigma-Aldrich, St. Louis, MO, USA). Then, 100 μL of MTT solution (0.5 mg/mL) was added to each well, and the cells were further incubated for an additional 4 h. The supernatant was removed, and the formazan was resolved with 1 mL/well of DMSO. The optical density was measured by a microplate reader (BioTek Instruments Inc, Winooski, VT, USA) at 540 nm.

### 4.6. Measurement of MMP-1 and Procollagen Type I Production

The production of MMP-1 (Abcam, Cambridge, UK) and pro-collagen type I (TaKaRa Bio Inc., Shiga, Japan) in cell culture media were quantified by ELISA kits according to the manufacturer’s instructions. MMP-1 or procollagen type I expressions are normalized to total protein amount.

### 4.7. Western Blot Analysis

Total cellular protein extracts from UVB-irradiated cells were prepared, as described previously [30]. Proteins were resolved by SDS-PAGE on 12% polyacrylamide gel and electrotransferred to nitrocellulose membrane. Immunoblot was incubated in blocking solution (5% BSA) and then with primary antibodies against p38, phospho-p38, ERK, phospho-ERK, c-Fos, phospho-c-Fos, α-tubulin (1:1000 dilution, Cell Signaling, Beverly, MA, USA), and JNK, phospho-JNK, c-Jun, phospho-c-Jun (1:1000 dilution, Santa Cruz Biotechnology Inc., Santa Cruz, CA, USA). For the secondary reaction, the membranes were washed and incubated with goat anti-rabbit or goat anti-mouse horseradish peroxidase-conjugated immunoglobulin G (IgG) secondary antibody. The protein expression level was measured by the enhanced chemiluminescence (ECL) detection system (Bio-rad, Hercules, CA, USA) and visualized using the ChemiDoc™ (Bio-rad, Hercules, CA, USA). The optical density of each representative blot was presented on the basis of the internal control protein (α-tubulin).

### 4.8. Quantitative Real-Time Polymerase Chain Reaction (qRT-PCR) Analysis

Total RNA was isolated from cells with Trizol reagent (Takara, Shiga, Japan) and RNeasy^®^ Mini Kit (QIAGEN, Hilden, German), according to the manufacturer’s instruction. cDNA was synthesized from 1 μg of total RNA using an iScript™ cDNA Synthesis Kit (Bio-Rad Laboratories Inc., Hercules, CA, USA). Then, the qRT-PCR analysis was performed with FastStart Essential DNA Green Master with LightCycler Real-Time PCR machine (Roche, Basel, Switzerland). The primer sequences used in qRT-PCR analysis were shown below.

Filaggrin forward 5′- AGT GCA CTC AGG GGG CTC ACA -3′ and Filaggrin reverse 5′- CCG GCT TGG CCG TAA TGT GT -3′; Involucrin forward 5′- TTG GTC AGT GAA GCG ATG AG -3′ and Involucrin reverse 5′- AGA TCT GTC TGC AGG GCT GT -3′; Loricrin forward 5′- TCA TAA GAA ACC CCG CTG AG -3′ and Loricrin reverse 5′- AAG GAA GGA GAG CCT GGA AG -3′; Caspase-14 forward 5′- CAA ACA CAT GGG TCA CTT GC -3′ and Caspase-14 reverse 5′- CAG AAC TGC TGA GCC TAC CC -3′; GAPDH forward 5′- GGA GCG AGA TCC CTC CAA AAT -3′ and GAPDH reverse 5′- GGC TGT TGT CAT ACT TCT CAT GG -3′. All results were normalized to internal control.

### 4.9. Statistical Analysis

All the experiments were repeated at least three times. The results are presented as the mean ± standard deviation (SD). Results were evaluated using the Statistical Analysis System (PRISM 5, Graph Pad). Analysis of variance (ANOVA) was used to identify statistically significant differences among the groups, and a *p*-value of 0.05 or less was considered statistically significant.

## 5. Conclusions

In conclusion, our current study showed that *Adenocaulon himalaicum* Edgew. extract (AHE) effectively protects skin photoaging by preventing collagen degradation through inhibiting MMP-1 via the MAPK/AP-1 signaling pathways. AHE significantly increased the expression of skin hydration factors, such as filaggrin, involucrin, loricrin, and caspase-14. Moreover, the newly identified neochlorogenic, as a component of AHE, ameliorated UVB-induced collagen deposition and reinforced skin hydration. Like the AHE-regulating mechanism, neochlorogenic acid modulated MAPK/AP-1 signaling molecules and skin barrier proteins. Taken together, these results suggest that AHE and neochlorogenic acid are well-qualified candidates for enhancing the conditions of photoaged skin.

## Data Availability

The data presented in this study are available on request from the corresponding author.

## Supplementary Materials

The following are available online at https://www.mdpi.com/article/10.3390/plants10081669/s1, Figure S1: ^1^H-NMR spectrum of neochlorogenic acid, Figure S2: ^13^C-NMR spectrum of neochlorogenic acid. Figure S3. Effect of AHE on MMP-1 mRNA expression levels in UVB-irradiated Hs68 fibroblasts, Figure S4. Effect of neochlorogenic acid on MMP-1 mRNA expression levels in UVB-irradiated Hs68 fibroblasts.
