# Supplementary material for: The Protective Effect of Adenocaulon himalaicum Edgew. and Its Bioactive Compound Neochlorogenic Acid against UVB-Induced Skin Damage in Human Dermal Fibroblasts and Epidermal Keratinocytes
Source: Plants (Basel). 2021 Aug 13;10(8):1669. doi: 10.3390/plants10081669 (PMC8399472; doi:10.3390/plants10081669)
Supplement: Supplementary file 1 [file plants-10-01669-s001.zip › plants-1322436-supplementary.pdf]

Supplementary materials

Protective Effect of *Adenocaulon himalaicum* Edgew. and Its Bioactive Compound Neochlorogenic Acid against UVB-Induced Skin Damage

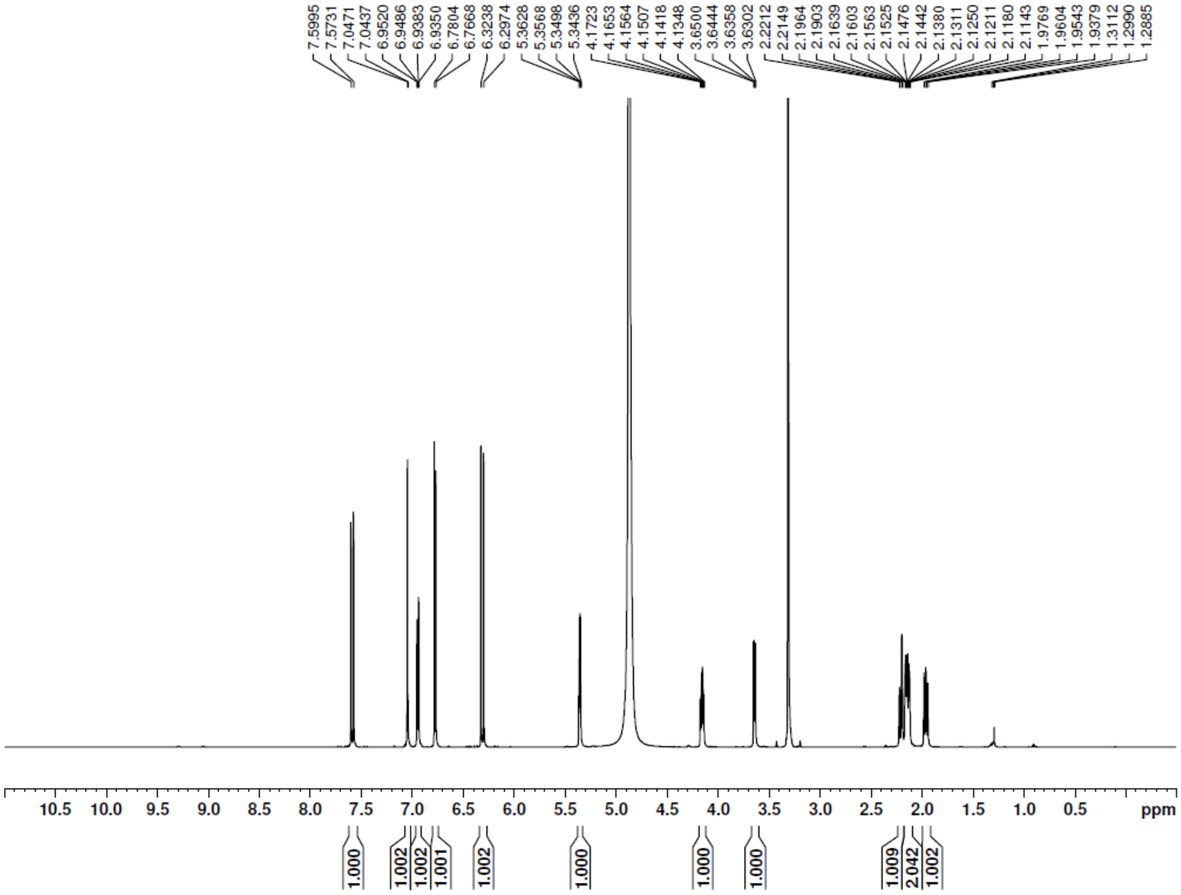

Figure S1. ^{1}H-NMR spectrum of neochlorogenic acid

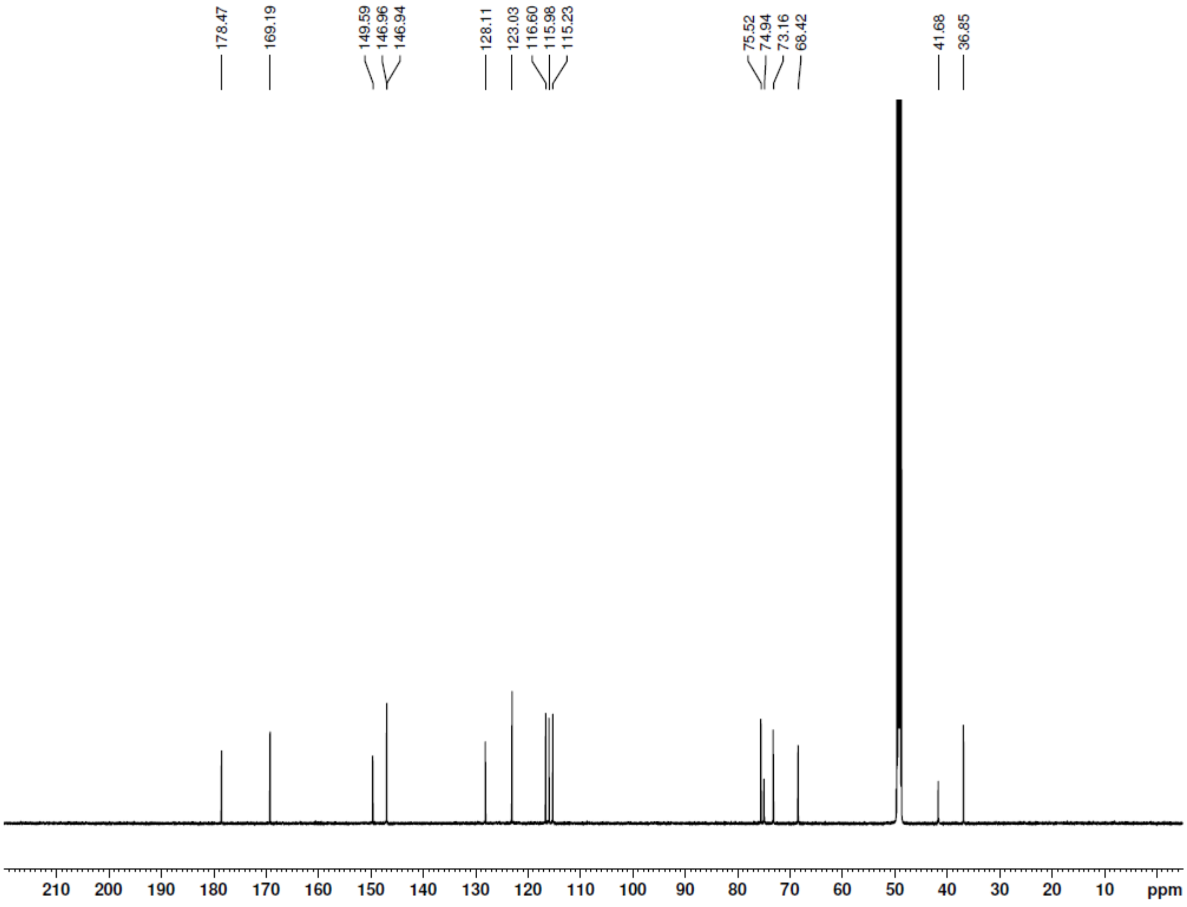

Figure S2. ^{13}C-NMR spectrum of neochlorogenic acid

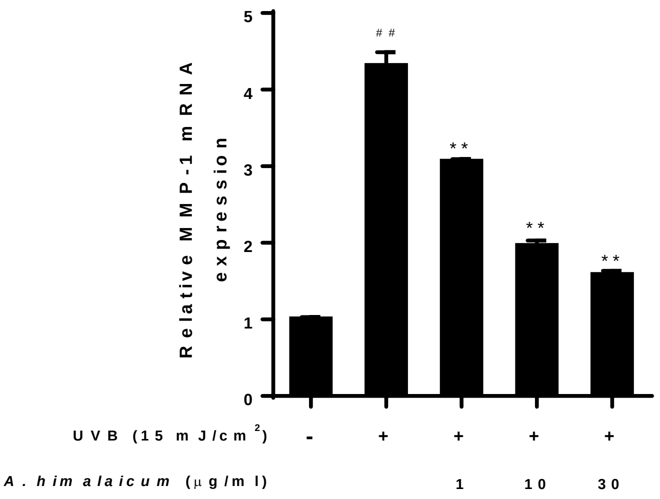

Figure S3. Effect of AHE on MMP-1 mRNA expression levels in UVB-irradiated Hs68 fibroblasts. Cells were exposed to UVB at 15 mJ/cm[2] and then treated with AHE (1, 10, 30 μg/ml) for 24 h. Total cellular RNA was extracted from AHE-treated cells. mRNA levels of MMP-1 were quantified by qRT-PCR and adjusted to GAPDH. Results are expressed as mean ± S.D. of three independent experiments; ##P<0.01 com-pared with the non-UVB irradiated control; **P<0.01 compared with the UVB-irradiated control.

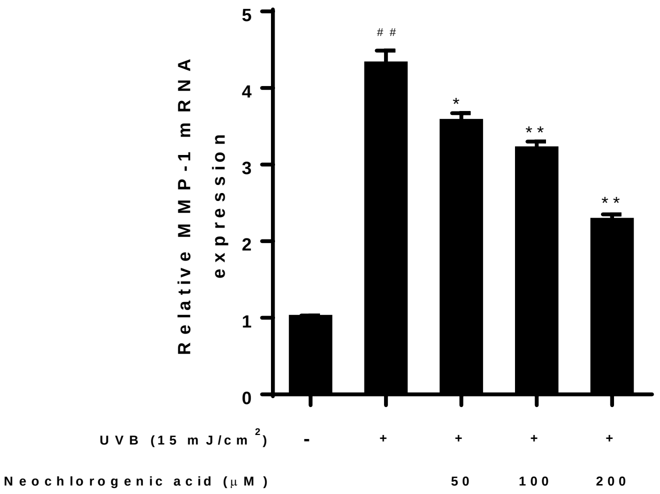

Figure S4. Effect of neochlorogenic acid on MMP-1 mRNA expression levels in UVB-irradiated Hs68 fibroblasts. Cells were exposed to UVB at 15 mJ/cm[2] and then treated with neochlorogenic acid (50, 100, 200 μM) for 24 h. Total cellular RNA was extracted from AHE-treated cells. mRNA levels of MMP-1 were quantified by qRT-PCR and adjusted to GAPDH. Results are expressed as mean ± S.D. of three independent experiments; ##P<0.01 com-pared with the non-UVB irradiated control; **P<0.05, **P<0.01

compared with the UVB-irradiated control.
